# Hydrogenated fat intake during pregnancy and lactation caused increase in TRAF-6 and reduced AdipoR1 in white adipose tissue, but not in muscle of 21 days old offspring rats

**DOI:** 10.1186/1476-511X-10-22

**Published:** 2011-01-25

**Authors:** Juliana L de Oliveira, Lila M Oyama, Ana Cláudia L Hachul, Carolina Biz, Eliane B Ribeiro, Claudia M Oller do Nascimento, Luciana P Pisani

**Affiliations:** 1 Departamento Fisiologia, Disciplina de Fisiologia da Nutrição, Universidade Federal de São Paulo, São Paulo, SP, Brasil; 2 Departamento de Ciências da Saúde, Universidade Federal de São Paulo, Santos, SP, Brazil

## Abstract

**Background:**

Although lipids transfer through placenta is very limited, modification in dietary fatty acids can lead to implications in fetal and postnatal development. *Trans *fatty acid (TFA) intake during gestation and lactation have been reported to promote dyslipidemia and increase in pro- inflammatory adipokines in offspring. The aim of this study was to evaluate whether the alterations on pro-inflammatory cytokines and dyslipidemia observed previously in 21-d-old offspring of rats fed a diet containing hydrogenated vegetable fat during gestation and lactation were related to alterations in TLR-4, TRAF-6 and adipo-R1 receptor in white adipose tissue and muscle. On the first day of gestation, rats were randomly divided into two groups: (C) received a control diet, and (T) received a diet enriched with hydrogenated vegetable fat, rich in *trans *fatty acids. The diets were maintained throughout gestation and lactation. Each mother was given eight male pups. On the 21st day of life the offspring were killed. Blood, soleus and extensor digital longus (EDL) muscles, and retroperitoneal (RET) white adipose tissue were collected.

**Results:**

21-d-old of T rats had higher serum triacylglycerols, cholesterol, and insulin. The Adipo R1 protein expression was lower in RET and higher in EDL of T group than C. TLR-4 protein content in all studied tissues were similar between groups, the same was verified in TRAF-6 protein expression in soleus and EDL. However, TRAF-6 protein expression in RET was higher in T than C.

**Conclusion:**

These results demonstrated that maternal ingestion of hydrogenated vegetable fat rich in TFAs during gestation and lactation decrease in Adipo R1 protein expression and increase in TRAF-6 protein expression in retroperitoneal adipose tissue, but not in skeletal muscle, which could contributed for hyperinsulinemia and dyslipidemia observed in their 21-d-old offspring.

## Introduction

Inadequate maternal nutrition during gestation and/or lactation can alter aspects of morphological and physiological development of pups, increasing the predisposition on the adult life to metabolic diseases, like diabetes mellitus and cardiovascular disease [1-3]. Fetal nutrition depends on the concentration of nutrients in maternal bloodstream, placental perfusion, and transfer of these nutrients through placenta [4-6].

During gestation, changes in the maternal metabolism occur in order to supply nutrition to the fetus. Lipids play a fundamental role in fetal development. Although lipids transfer through placenta is very limited, changes in dietary fatty acids can lead to implications in fetal and postnatal development [7].

Several studies verified that consumption of high amounts of *trans *fatty acid (TFA) increases blood total cholesterol, VLDL, LDL-cholesterol and triglycerides, and decreases blood HDL-cholesterol, which as consequence increases the chances of development of metabolic syndrome. Also, [8] demonstrated that treatment with TFA has a much greater effect in decreasing adipocyte insulin sensitivity than treatment with saturated fatty acids. The authors explained these results in part by a reduction of plasma membrane fluidity in the rats treated with TFA.

In these sense, we have previously showed dyslipidemia and increased body fat, high TNF-α and PAI-1 mRNA levels, and low plasma and mRNA adiponectin levels in 21-d-old offspring of rats fed a diet containing hydrogenated vegetable fat during gestation and lactation [9].

Insulin resistance and type 2 diabetes mellitus, obesity and heart disease [10,11] have been associated with decrease in adiponectin serum levels. Several studies have been demonstrated that adiponectin reduces hepatic production of glucose and the concentration of triglycerides in the muscles, thus ameliorating insulin sensitivity [12].

Additionally, TFA in the diet was found to increase the production of pro-inflammatory cytokines, like IL-6 and TNF-α [13-20]. These increase could be regulated by Toll-like receptor 4 (TLR-4) signaling, which stimulates inflammatory cytokine production [21].

Toll-like receptors (TLR) are trans-membrane proteins that play an important role in recognizing microbial pathogens and mediating whole body inflammation [22]. Tsukumo et al [23] demonstrated that C3H/HeJ mice, which have a loss-of-function mutation in TLR4, did not develop obesity and insulin resistance induced by high fat diet. TLR4 is a subclass of TLRs that can be activated by lipopolysaccharide (LPS) and by nonbacterial agonists, such as saturated fatty acids [24,25]. The activation of TLR4 signaling induces upregulation of intracellular inflammatory pathways related to the induction of insulin resistance, each included the adaptor molecule myeloid differentiation primary-response protein 88 (Myd88), IL-1R-associated kinases (IRAKs), transforming growth factor-β (TGF-β-) activated kinase (TAK1), TAK-1 binding protein (TAB1 and 2), and tumor necrosis factor (TNF)-receptor-associated-factor-6 (TRAF-6) [26,27].

The aim of this study was to evaluate whether the alterations on pro-inflammatory cytokines and dyslipidemia observed previously in 21-d-old offspring of rats fed a diet containing hydrogenated vegetable fat during gestation and lactation were related to alterations in TLR-4, TRAF-6 and adipo-R1 receptor in white adipose tissue and muscle.

## Materials and methods

### Animals and treatments

The experimental research committee of the Universidade Federal de São Paulo approved all procedures for the care of the animals used in this study. Rats were kept under controlled conditions of light (12-h light/12-h dark cycle with lights on at 0700 h) and temperature (24 ± 1°C). Three-month-old female Wistar rats were left overnight to mate, and copulation was verified the following morning by the presence of sperm in vaginal smears. On the first day of gestation, rats were isolated in individual cages and randomly divided into two groups, receiving a control diet (C diet, C group) or a diet enriched with hydrogenated vegetable fat (T diet, T group). The diets were maintained throughout gestation and lactation. On the day of delivery, considered day 0 of lactation, litters were adjusted to eight pups each. The pups were weighed at 21 of lactation, when they were killed.

Both diets were prepared according to the recommendations of the American Institute of Nutrition (AIN-93G) [28] being similar in calories and lipid content. The source of lipids for the C diet was soybean oil, and the principal source for the T diet was partially hydrogenated vegetable fat, rich in TFAs. The centesimal composition of the diets is presented in Table 1. The fatty acid profile of each diet, was previously described by Pisani et al [9].

**Table 1:** Composition of control and TFA-enriched diets according to AIN-93

Ingredient	Diet (g/100 g)	
	**C**	**T**

Casein*	20	20

L-cystine^†^	0.3	0.3

Cornstarch^†^	62	62

Soybean oil^‡^	8	1

Hydrogenated vegetable fat^§^	-	7

Butyl hydroquinone^†^	0.0014	0.0014

Mineral mixture	3.5	3.5

Vitamin mixture^¶^	1.0	1.0

Cellulose^†^	5.0	5.0

Choline bitartrate^†^	0.25	0.25

Energy (kcal/g)	4.0	4.0

C, control; T, hydrogenated vegetable fat; TFA, *trans*-fatty acid

* Casein was obtained from CoperLab, São Paulo, Brazil.

^† ^L-cystine, cornstarch, butyl hydroquinone, cellulose and choline bitartrate were obtained from Viafarma, São Paulo, Brazil.

^‡ ^Oil was supplied from soybean (Lisa/Ind. Brazil).

^§ ^Hydrogenated vegetable fat was supplied from Unilever, São Paulo, Brazil.

|| Mineral mix provided (mg/kg) calcium 5000, phosphorus 1561, potassium 3600, sodium 1019, chloride 1571, sulfur 300, magnesium 507, iron 35, copper 6.0, manganese 10.0, zinc 30.0, chromium 1.0, iodine 0.2, selenium 0.15, fluoride 1.00, boron 0.50, molybdenum 0.15, silicon 5.0, nickel 0.5, lithium 0.1, vanadium 0.1 (AIN-93G mineral mix DYETS 210025, Dyets Inc., Bethlehem, PA, USA).

^¶ ^Vitamin mix (mg/kg diet) provided thiamin HCL 6.0, riboflavin 6.0, pyridoxine HCL 7.0, niacin 30.0, calcium pantothenate 16.0, folic acid 2.0, biotin 0.2, vitamin B12 25.0, vitamin A palmitate 4000 IU, vitamin E acetate 75, vitamin D3 1000 IU, vitamin KI 0.75 (AIN-93G, vitamin mix, DYETS 310025, Dyets Inc.).

### Experimental procedures

After 21 d of lactation, the offspring were decapitated. Trunk blood was collected and immediately centrifuged. Serum was separated and stored at -70°C for later determination of triacylglycerols, cholesterol, HDL- cholesterol, glucose, insulin and adiponectin. The retroperitoneal (RET) white adipose tissue, soleus and extensor digital longus (EDL) were dissected, immediately frozen in liquid nitrogen, stored at -70°C, and used for quantification of TLR4, TRAF6 and AdipoR1 mRNA and protein expression.

### Carcass lipid and protein content

Other groups of C and T rats were used for determination of carcass lipid and protein content. Carcasses were eviscerated, weighed, and stored at -20°C. Lipid content was measured as described by Stansbie et al. and standardized using the method described by Oller do Nascimento and Williamson, 1986 [29]. Briefly, the eviscerated carcass was autoclaved at 120°C for 90 min and homogenized with double the mass of water. Triplicate aliquots of this homogenate were weighed and digested in 3 mL of 30% KOH and 3 mL of ethanol for ≥2 h at 70°C in capped tubes. After cooling, 2 mL of 12 N H_2_SO_4 _was added and the sample washed three times with petroleum ether for lipid extraction. Results are expressed as grams of lipid per 100 g of carcass. For protein measurements, aliquots of the same homogenate (approximately 1 g) were heated to 37°C for 1 h in 0.6 N KOH with constant shaking. After clarification by centrifugation, protein content was measured according to the method described by Lowry et al [30].

### Biochemical and hormonal serum analyses

Glucose, triacylglycerols, total cholesterol, and HDL- cholesterol serum concentrations were measured by an enzymatic colorimetric method using commercial kits (Labtest, Brazil). Insulin and adiponectin concentrations were quantified using specific enzyme-linked immunosorbent assay kits (Linco Research, USA).

### RNA extraction and real-time polymerase chain reaction

Total RNA from the tissues was extracted with Tri-Reagent (Sigma, St. Louis, MO, USA), and its concentration was determined from absorbance at 260 nm.

The TLR4 and AdipoR1 mRNA from RET, soleus and EDL were quantified by real-time polymerase chain reaction. RNA samples were previously treated with DNAse (DNA-free, Ambion, Austin, TX, USA). One microgram of each sample was reverse transcribed using an M-MLV Reverse Transcriptase kit (Promega, Madison, WI, USA), and cDNA was synthesized in a final volume of 50 μL. Relative levels of TLR4 and AdipoR1 mRNA were quantified in real time, using a SYBR Green primer in an ABI Prism 7500 Sequence Detector (both from Applied Biosystems, Foster City, CA, USA). Relative levels of the housekeeping gene hypoxanthine phosphoribosyltransferase were measured. The primers used were: AdipoR1 5'-CTT CTA CTG CTC CCC ACA GC-3' (sense) and 5'-TCC CAG GAA CAC TCC TGC TC-3' (antisense); and TLR-4 5'-GCATCATCTTCATTGTCCTTGAGA-3' (sense) and 5'-CTACCTTTTCGGAACTTAGGTCTACT-3' (antisense); and hypoxanthine phosphoribosyltransferase: 5'-CTCATGGACTGATTATGGACAGGA-3' (sense) and 5'-GCAGGTCAGCAAAGAACTTATAGC-3' (antisense).

Results were obtained using Sequence Detector software (Applied Biosystems) and are expressed as a relative increase, using the method of 2^-ΔΔCt ^described by Livak and Schmittgen [31].

### Protein analysis by Western Blotting

After euthanasia, the RET, soleus and EDL muscle were rapidly removed, homogenized in 1.0 ml extraction buffer (100 mM Trizma, 1% SDS, 100 mM sodium pyrophosphate, 100 mM sodium fluoride, 10 mM EDTA and 10 mM sodium orthovanadate) and boiled for 10 min. The extracts were then centrifuged at 12,000 rpm at 4°C for 40 min to remove the insoluble material. Protein determination in the supernatants was performed by the Bradford dye method using the Bio-Rad reagent (Bio-Rad Laboratories, Hercules, CA, USA). The proteins were treated with Laemmli sample buffer containing dithiothreitol and boiled for 5 min before loading onto 8% SDS-PAGE in a Bio-Rad miniature slab gel apparatus.

Electrotransfer of proteins from the gel to the nitrocellulose was performed for 1 h at 120 V (constant) in a Bio-Rad miniature transfer apparatus. Nonspecific protein binding to the nitrocellulose was reduced by pre-incubation for 1 h at 22°C in blocking buffer (5% non-fat dry milk, 10 mM Tris, 150 mM NaCl and 0.02% Tween 20). The nitrocellulose membranes were incubated overnight at 4°C with antibodies against TLR4, TRAF6, AdipoR1 and α-Tubulin obtained from Santa Cruz Biotechnology (Santa Cruz, CA, USA), diluted in blocking buffer combined with 1% bovine serum albumin (BSA) and then washed for 30 min in blocking buffer without BSA. The blots were subsequently incubated with a peroxidase-conjugated secondary antibody for 1 h at 22°C and processed for enhanced chemiluminescence to visualize the immunoreactive bands. Band intensities were quantificated by optical densitometry (Scion Image-Release Beta 3b, NIH, USA) of the developed autoradiographs.

### Statistical analysis

All results are presented as mean ± standard error of the mean. Statistical significances of the differences between the means of the two groups of samples were assessed using Student's *t *test. Differences were considered to be statistically significant at *P *< 0.05.

## Results

Body weight of the pups did not differ between the C and T groups at 21 d of life. However, the T diet promoted a significant increase in RET relative weight and in carcass lipid content (p < 0.054) with no difference in carcass protein content. Also, T diet increased serum concentrations of triacylglycerols, total cholesterol and insulin (Table 2).

**Table 2:** Body weight (g), retroperitoneal white adipose tissue (RET), soleus and EDL muscle relative weights (g/100g b. w.), carcass protein and fat content (g per 100 g of body weight), serum glucose, triacylglycerols, cholesterol, HDL cholesterol, insulin, and adiponectin in the C and T groups.

	Control	Trans
Body weight (g)	41.55 ± 2.49	40.50 ± 1.79

RET relative weight (g/100g)	0.21 ± 0.03	0.27 ± 0.01*

Soleus relative weight (g/100g)	0.09 ± 0.006	0.09 ± 0.003

EDL relative weight (g/100g)	0.09 ± 0.007	0.10 ± 0.004

Carcass lipid content (g/100g)	3.98 ± 0.34	4.61 ± 0.228

Carcass protein content	10.38 ± 1.12	11.536 ± 0.29

Triacylglycerol (mg/dL)	201.82 ± 20.75	456.93 ± 25.50*

Glucose (mg/dL)	90.46 ± 3.42	99.77 ± 3.43

Total cholesterol (mg/dL)	146.86 ± 3.4	171.22 ± 7.47*

HDL-cholesterol (mg/dL)	34.49 ± 1.68	38.46 ± 6.93

Adiponectin (μg/mL)	9.87 ± 1.35	11.52 ± 1.94

Insulin	0.63 ± 0.05	1.07 ± 0.19*

Data are means ± SEMs of 10 determinations/group. * *P *< 0.05 versus C.

### Adipo-R1 gene and protein expression

**Figure 1:**
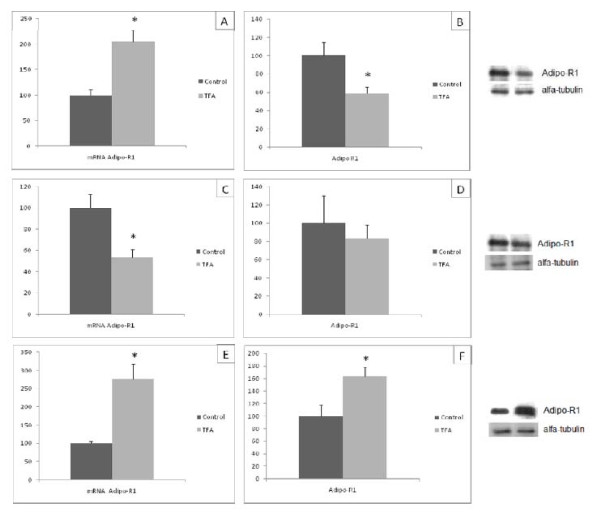
**Adipo-R1 mRNA (A,C,E) and protein quantification (B,D,F) in retroperitoneal white tissue (A and B), soleus muscle (C and D) and EDL (E and F) of the C and T groups**. Data are means ± SEMs of 5-9 determinations per group. Results are expressed in arbitrary units, stipulating 100 as the control value. *P < 0.05 versus C.

Adipo-R1 mRNA levels in the RET and EDL of group T were 2 and 2.75 times higher than in group C, respectively (Figure 1A, E). In contrast, in soleus muscle Adipo R1 mRNA levels were 45% lower in group T (Figure 1C). The Adipo R1 protein expression was lower in RET and higher in EDL of T group than C (Figure 1B, F, respectively). Quantification of Adipo-R1 in soleus produced similar figures in both groups (Figure 1D).

### TLR-4 gene and protein expression

**Figure 2:**
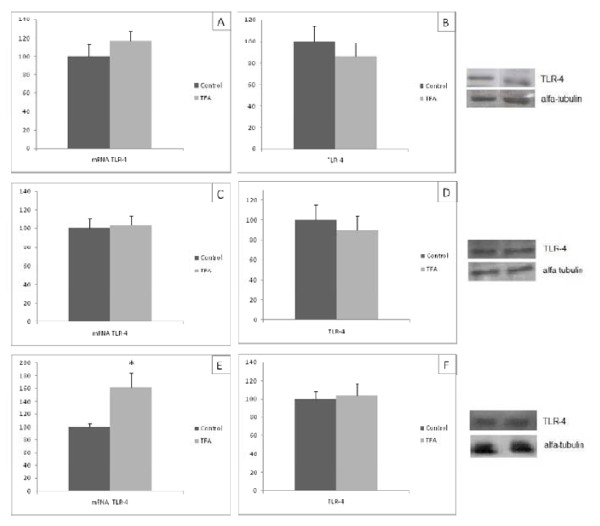
**TLR-4 mRNA (A,C,E) and protein quantification (B,D,F) in retroperitoneal white tissue (A and B), soleus muscle (C and D) and EDL (E and F) of the C and T groups**. Data are means ± SEMs of 5-9 determinations per group. Results are expressed in arbitrary units, stipulating 100 as the control value. *P < 0.05 versus C.

TLR-4 mRNA levels in the soleus of group T were 1.6 times higher than in group C (Figure 2E). Quantification of TLR-4 mRNA in RET and EDL produced similar figures in both groups (Figure 2A, C, respectively). The ingestion of diet containing trans fatty acid during lactation and gestation did not modified the TLR-4 protein content in all studied tissues from pups with 21 d of life (Figure 2B, D, F).

### TRAF- 6 protein expression

**Figure 3:**
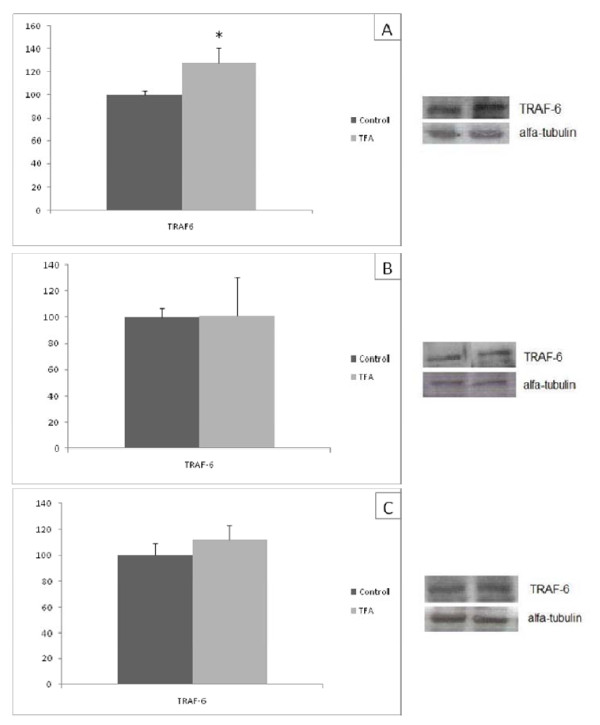
**TRAF-6 protein quantification in retroperitoneal white tissue (A), soleus muscle (B) and EDL (C) of the C and T groups**. Data are means ± SEMs of 5-9 determinations per group. Results are expressed in arbitrary units, stipulating 100 as the control value. *P < 0.05 versus C.

The ingestion of hydrogenated vegetable fat during gestation and lactation caused an increase in TRAF-6 protein expression in RET of 21 day old pups (Figure 3A). However, the TRAF-6 protein expression was similar in soleus and EDL between C and T groups (Figure 3 B, C).

## Discussion

In this study, maternal ingestion of hydrogenated vegetable fat rich in TFAs during gestation and lactation altered the blood lipid profiles, increased insulin serum levels accompanied by a decrease in Adipo R1 protein expression and increase in TRAF-6 protein expression in retroperitoneal adipose tissue, which could contributed for insulin resistance and the dyslipidemia observed in their 21-d-old offspring.

The body weight of offspring exposed to maternal TFAs was similar to those of the C group, confirming our previous findings [9,32]. Also, Colandré et al. [33] found that the ingestion of TFAs by adult rats for 30 d failed to change weight gain. On the other hand, the ingestion of TFA reduced body weight gain in 90-d- old offspring [34] or increased this parameter in 14- and 45-d-old rats exposed during lactation and after weaning to a normolipidic diet of which 7% was hydrogenated fat [35]. These results suggest that the effect on body weight of dietary TFAs depends on the period of life that the animal is exposure.

In the present study we analyzed the lipid content in the carcass without any viscera and visceral fat pad, just to quantify the subcutaneous fat pad which was similar between T and C groups. Previously, we have found an increase in carcass total lipid content (with all fat pads) in group T comparing to C [9]. In fact, in the present study the RET relative weight was higher in T than C group. Taken all together these results demonstrated that the maternal ingestion of hydrogenated vegetable fat rich in TFAs during gestation and lactation promoted an increase in visceral fat pad accumulation in the 21-d-old offspring. It could be suggested that this occur, at least partially, by a decrease in adiponectin effect on RET since the adipoR1 was lower in retroperitoneal adipose tissue from T than C. It has been shown that adiponectin attenuates acetyl-CoA carboxylase activity reducing lipid synthesis and indirectly enhances fatty acid oxidation by blocking the production of malonyl-CoA [36]. In this sense, Silva et al. [35] studying the effects of TFA ingestion just during lactation, verified increased in de novo lipogenesis rates and lipid contents in the EPI adipose tissue of offspring aged 45 d.

In accordance to our formerly report [9], feeding lactating rats with a diet 7% of which was made up of hydrogenated vegetable fat promoted an increase in serum triacylglycerol and total cholesterol levels. TFAs ingestion during lactation led to increased milk content of, long-chain saturated fatty acids, and total lipid, and decreased polyunsaturated fatty acids [37], and TFAs and saturated fatty acids increased VLDL secretion [38]. Ng et al. [39], reported that triacylglycerol concentration is inversely correlated with adiponectin plasma concentration. Then, it is possible that the decrease in adiponectin receptor in RET of T group could be an important mechanisms to contributed to the high serum triacylglycerol and cholesterol levels in this group. A similar effect was induced by administration of TNF-α to hamsters [40]. Maternal ingestion of hydrogenated vegetable fat rich in TFAs during gestation and lactation elevated TNF-α gene expression in RET of 21-d- old offspring, and it has been previously shown that TNF-α gene expression in adipose tissue correlates with circulating TNF-α levels [41]. Thus, high TNF-α activity is likely to have contributed to dyslipidemia observed in T group. Alteration of plasma lipoprotein profile, with increased risk of cardiovascular diseases, decreased insulin sensitivity, and higher risk of type 2 diabetes, has been reported to follow the long-term intake of TFAs [16,42-44]. However, other studies have failed to find an effect of TFAs hyperinsulinemia and insulin resistance [9,45,46].

In the present study we have shown that maternal ingestion of trans fatty acid caused an increase in insulin serum level in 21-d-old offspring accompanied by an increase in TRAF-6 and decrease in AdipoR1 protein expression in RET regardless of a decrease in AdipoR1 mRNA level. Recent study reported decreased in AdipoR1 protein levels in monocytes from type 2 diabetic subjects, while AdipoR1 mRNA content was actually increased, reinforcing that the changes in protein do not always occur in parallel to change in mRNA levels [47].

Previously, it has been shown an increase in protein expression of TLR-2, MyD88, and TRAF6 as well as NF-κB in human adipose tissue in states of obesity and diabetes mellitus type 2 [48]. These results suggested that the insulin resistance caused by *trans *fat acid ingestions could be related to the induced innate immune cascade in adipose tissue promoting an increase in pro-inflammatory adipokines expression, as reported by our group [9,34] and reducing the adiponectin effect by reducing the adipoR1 protein expression. Activation of proinflammatory adipokines in adipose tissue is coordinated through NF-κB, a key transcription factor in the inflammatory cascade. The increase in TRAF-6, observed in the present study contributes in inflammatory responses leading, probably, the activation of the NF-κB pathway. We did not measure the NF-kB DNA binding, however the increase in TRAF-6 normally is related to activation of NF-κB pathway [49].

Creely et al [48] observed a positive correlation between insulin serum concentration and endotoxinemia. Then it is possible to suggest that the hyperinsulinemia, present in TFA group, associated to the effect of fatty acids in the intestinal microbial composition [50] modified the intestinal permeability elevating lipopolysaccharides activating the inflammatory cascade, promoting an increase in TRAF-6.

The TFA ingestion during gestation and lactating caused a different effect on oxidative muscle (soleus) and glycolytic muscle (EDL). Different type of fiber shows different response, for instance expression of TNF-*α *has been reported in glycolytic fibers from sedentary healthy subjects, but not in oxidative fibers [51].

The TFA did not modify the parameters analyzed in soleus muscle, however promoted an increase in AdipoR1 protein expression and in TLR-4 gene expression in EDL of 21-d-offspring. As previously reported AdipoR1 protein content in soleus muscle was not affected either by saturated or polyunsaturated fatty acid rich diet [52]. Despite of several studies demonstrated that fatty acid rich diet could promote an insulin resistance in muscle, our results suggest that the change in AdipoR1 protein in muscle is not important for impairing inflammatory response in muscle or that the TFA during gestation and lactation caused more important modification in adipose tissue than muscle related to the inflammatory process.

In conclusion the present study confirmed that maternal ingestion of hydrogenated vegetable fat rich in TFAs during gestation and lactation altered the blood lipid profiles of 21-d old-offsprig. Also, demonstrated that caused an increase in insulin serum levels accompanied by a decrease in Adipo R1 protein expression and increase in TRAF-6 protein expression in retroperitoneal adipose tissue, but not in skeletal muscle, which could contributed for hyperinsulinemia and dyslipidemia observed in their 21-d-old offspring.

## Competing interests

The authors declare that they have no competing interests.

## Authors' contributions

JLO designed the study, carried out the experiments, performed the statistical analysis and drafted the manuscript.

LMO participated in the design of the study helped to carried out the experiments.

ACLH helped to carried out the experiments.

CB helped to carried out the experiments.

EBR revised and helped to draft the manuscript.

CON help to conceive the study, participated in its design, and helped to draft the manuscript.

LPP conceived of the study, participated in its design, coordination and helped to draft the manuscript.

All authors read and approved the final manuscript
